# A crosstalk between circular RNA, microRNA, and messenger RNA in the development of various brain cognitive disorders

**DOI:** 10.3389/fnmol.2022.960657

**Published:** 2022-10-18

**Authors:** Liang He, Furong Zhang, Yuling Zhu, Meilin Lu

**Affiliations:** ^1^Department of Anesthesiology, Yan'an Hospital of Kunming City, Kunming Medical University, Kunming, China; ^2^Department of Anesthesiology, The First Affiliated Hospital of Kunming Medical University, Kunming, China

**Keywords:** circular RNAs, cognitive dysfunction, neurocognitive, network, ceRNA

## Abstract

Patients with Alzheimer's disease (AD), Parkinson's disease (PD), traumatic brain injury (TBI), stroke, and postoperative neurocognitive disorder (POND) are commonly faced with neurocognitive disorders with limited therapeutic options. Some non-coding ribonucleic acids (ncRNAs) are involved in the development of various brain cognitive disorders. Circular RNAs (circRNAs), a typical group of ncRNAs, can function as competitive endogenous RNAs (ceRNAs) to dysregulate shared microRNAs (miRNAs) at post-transcription level, inhibiting regulation of miRNAs on their targeted messenger RNAs (mRNAs). circRNAs are abundant in central nervous system (CNS) diseases and cause brain disorders, but the exact roles of circRNAs are unclear. The crosstalk between circRNA, miRNA, and mRNA plays an important role in the pathogenesis of these neurocognitive dysfunction diseases and abnormal conditions including AD, PD, stroke, TBI, and POND. In this review, we summarized the participation of circRNA in neuroglial damage and inflammation. Finally, we aimed to highlight the regulatory mechanisms of circRNA–miRNA–mRNA networks in the development of various brain cognitive disorders and provide new insights into the therapeutics of these diseases.

## Introduction

Microribonucleic acids (miRNAs) are small, non-coding, single-stranded linear RNAs spanning an average of 22 nucleotides in length. miRNAs can bind to the 3′ untranslated region (UTR) of messenger RNAs (mRNAs) *via* miRNA recognition elements (MRE) of target genes, which are presented in repression or degradation at the posttranscriptional levels, resulting in the dysregulation of the expression of target proteins (Salmena et al., 2011; Salim et al., 2022). Unlike classical linear RNAs, circular RNAs (circRNAs) are a novel group of evolutionarily conserved non-coding RNAs (ncRNAs) that form covalently closed continuous loop structures without 5' caps or 3' Poly A tails and act as sponges of miRNAs (Kristensen et al., 2019; Chen et al., 2022). Here, a total of 262,782 circRNAs have been presented in CIRCpedia v2 embracing over 180 RNA-seq data sets that cross six different species (Dong et al., 2018). Meanwhile, the crosstalk mechanism between circRNA, miRNA, and mRNA is not clearly understood.

**Figure 1:**
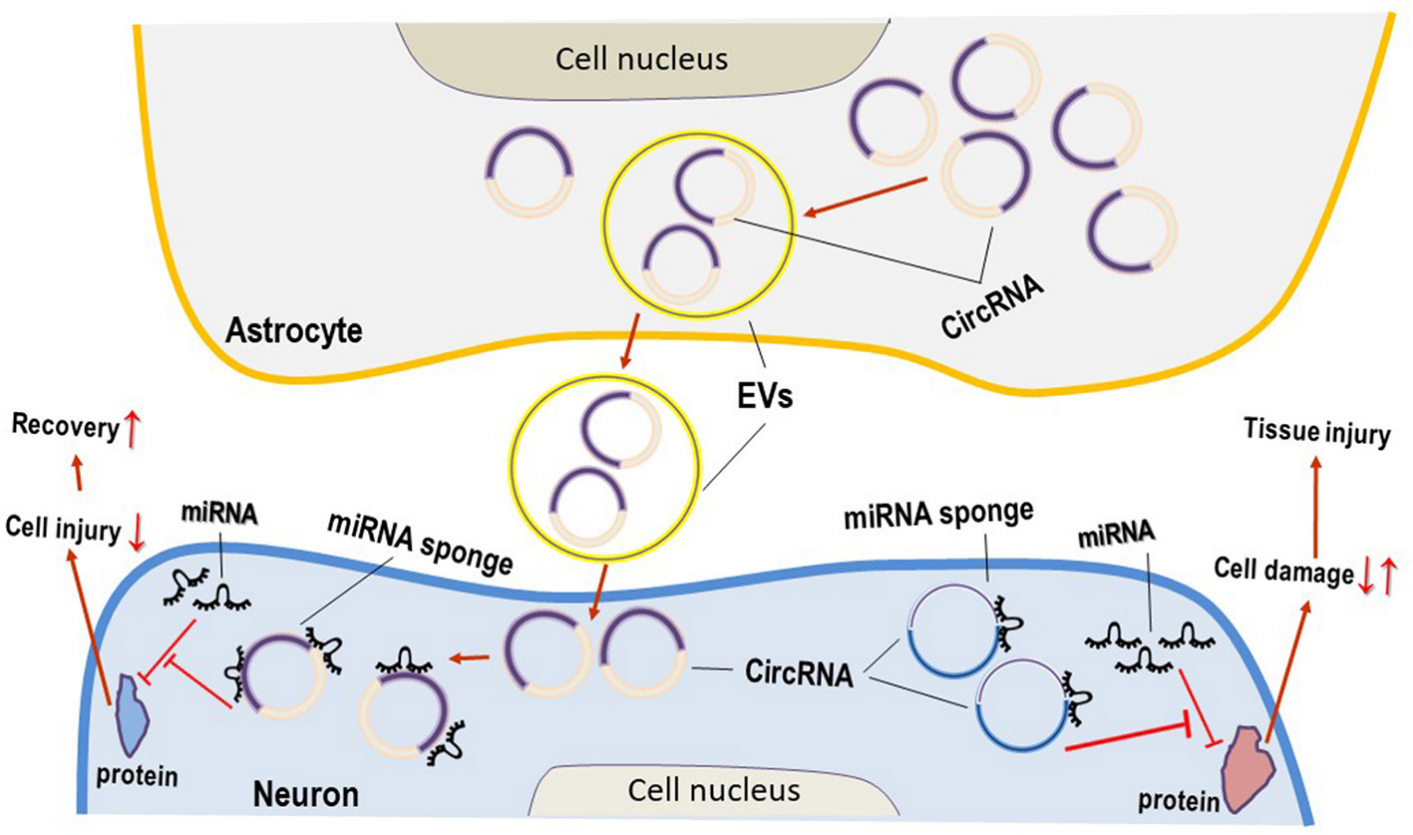
Circular RNAs (CircRNAs) act as the sponges of microRNAs (miRNAs) in neuroglial damage. Main circRNAs can act as miRNA sponges, resulting in the inhibition of miRNA on its target genes and/or proteins, and function as important roles in neuronal damage. Some circRNAs derived from extracellular vesicles (EVs) in astrocytes are beneficial for the attenuation of neurological disorders.

As mentioned above, most genomes are presented in a miRNA-dependent repression manner and are densely covered in MREs like the letters of an “RNA language,” which function as the entire mRNA dimension by identifying competitive endogenous miRNA (ceRNA) networks in the ncRNA pool (Salmena et al., 2011; Hansen et al., 2013). The ceRNA of long ncRNA in miRNA regulation is validated (Salmena et al., 2011). Some circRNAs could function as molecular sponges for other RNA transcripts through their miRNA binding sites by ceRNA (Hansen et al., 2013). circRNA functions as miRNA sponge to dysregulate stability, transcription, and translation of mRNAs (Figure 1), which are the target genes of miRNAs, in complicated mechanisms (Hansen et al., 2013; Yesharim et al., 2021; D'Anca et al., 2022).

A lot of studies indicate that the crosstalk between circRNAs, miRNAs, and mRNA can participate in many pathophysiological processes in the development of cerebral diseases (or abnormal conditions), such as Alzheimer's disease (AD) (Lu et al., 2019; Li et al., 2022), Parkinson's disease (PD) (Wang et al., 2021; Liu et al., 2022), traumatic brain injury (TBI) (Huang X. J. et al., 2022; Zheng et al., 2022), stroke (Dai et al., 2021; Xu T. et al., 2022), and postoperative neurocognitive disorder (POND) (He et al., 2021; Wu et al., 2021; Zhang M. X. et al., 2022). However, they have failed to manage the aforementioned brain cognitive disorders with effective therapeutics and to uncover largely unknown underlying mechanisms.

In this review, we discussed the effects of circRNAs on various brain neurocognitive disorders as mentioned above. We also found a network of circRNA–miRNA–mRNA for the potential treatment or therapeutics of the aforementioned neurocognitive impaired diseases or conditions.

## CircRNA–miRNA–mRNA networks in brain cognitive disorders

### Alzheimer's disease

Alzheimer's disease, the most common cause of dementia, is a rising global health issue with tremendous implications. A crucial characteristic of AD is an aberrant accumulation of β-amyloid (Aβ) peptides in the brain, in terms of enhanced cleavage of Aβ precursor protein (APP) by the rate-limiting β-site APP-cleaving enzyme 1 (BACE1) (Shi et al., 2017). The circRNA cerebellar degeneration-related protein 1 antisense (CDR1as, also known as ciRS-7), a gigantic molecule, functions as a miR-7 sponge or inhibitor (Kumar et al., 2017; Li et al., 2019). However, ciRS-7 expression inhibits the translation of nuclear factor kappa B (NF-κB) and induces its cytoplasmic localization, derepressing the expression of ubiquitin carboxyl-terminal hydrolase L1 (UCHL1), which enhances the degradation of APP and BACE1 so as to be available in neuroprotection (Shi et al., 2017). The downregulation of ciRS-7 upregulates miR-7 and lowers the expression of ubiquitin-conjugating enzyme E2A (UBE2A, a location in chr Xq24), which coordinates the clearance of Aβ *via* proteolysis and contributes to the accumulation of Aβ and the formation of senile plaque deposits (Zhao et al., 2016). However, the trend toward change of consistency relationship between CDR1as/ciRS-7 and miR-7 is still controversial. In addition, in terms of sponging or inhibition of miR-7, ciRS-7 may prevent miR-7 from degradation (Kleaveland et al., 2018).

Serum circHDAC9 was decreased in patients with AD and individuals with mild cognitive impairment. circHDAC9 acts as a miR-138 sponge, decreasing miR-138 expression and reversing the suppression of silent information regulator 1 (Sirt1) and excess production of Aβ in AD (Lu et al., 2019).

Circ_0000950 sponging miR-103 increases the expression of prostaglandin-endoperoxide synthase 2 (PTGS2) and elevates the levels of inflammatory cytokines [interleukin-1β (IL-1β), IL-6, and tumor necrosis factor α (TNF-α)] to inhibit neurite outgrowth and promote neuron apoptosis in AD (Yang et al., 2019). The downregulation of circ_0001588 upregulates miR-211-5p and induces the Sirt1/nuclear factor erythroid 2-related factor 2 (Nrf2)/heme oxygenase-1 (HO-1) pathway to prevent oxidative stress in AD (Zhu et al., 2020). CircAXL decreases miR-328 to upregulate the expression of BACE1 in AD (Li et al., 2022). Circ_0003611 downregulates miR-885-5p to aggravate Aβ-induced neuronal injury in AD (Pan et al., 2022). Circ_0005835 promotes the development of AD *via* regulating miR-576-3p expression (Xu X. et al., 2022).

The networks of circRNA–miRNA–mRNA are involved in mitogen-activated protein kinase (MAPK), mammalian target of rapamycin (mTOR), AMP-activated protein kinase (AMPK), and Wnt signaling pathways in the pathogenesis of AD (Li Y. et al., 2020). Certainly, in the presence of RNA-seq and prediction tools for circRNA bioinformatic analysis, the circRNA–miRNA–mRNA network will indicate one more potential role of epigenetic control over the expression of pathogenic genes in the human central nervous system (CNS) in the development and therapeutic treatment of sporadic AD.

### Parkinson's disease

Parkinson's disease, characterized by motor and non-motor symptoms, is also a neurodegenerative disorder. A growing body of research in recent decades has denoted that cognitive decline is a common non-motor symptom of PD (Hall and Lewis, 2019; Leng et al., 2020). With the development of potentially effective treatment, useful biomarkers (such as α-synuclein and some possible circRNAs) of PD may provide early diagnosis, therapeutic monitoring, and prognosis (Hosaka et al., 2019; Liu et al., 2022). The RNA-binding protein FUS acts as a new regulator of circRNA production in motor neurons in mice (Errichelli et al., 2017; D'Anca et al., 2022).

In PD, the neuroprotective role of miR-7 is induced through the alleviation of the suppression of NF-κB by lowering the expression of RelA (a component of NF-κB) due to dopaminergic neurotoxicity (Choi et al., 2014). In addition, miR-7 can target and downregulate α-synuclein, protecting neurons from oxidative stress (Junn et al., 2009; Zhu et al., 2021). circzip-2 sponging miR-60 decreases the expression of protein α-synuclein in the *Caenorhabditis elegans* model of PD (Kumar et al., 2018). In a mouse model, the circ_0003292/miR-132/Nr4a2 network may be involved in the molecular mechanism of PD (Jia et al., 2020).

In *in vivo* and *in vitro* studies, circRNA DLGAP4 (circDLGAP4) exerts a promotion in viability, a reduction in apoptosis, a decline in mitochondrial damage, and an enhancement in autophagy *via* modulating the miR-134-5p/CREB pathway in PD (Feng et al., 2020). circSAMD4A participates in the apoptosis and autophagy of dopaminergic neurons *via* the miR-29c-3p-mediated AMPK/mTOR pathway in PD (Wang et al., 2021). circPank1 promotes dopaminergic neuron neurodegeneration through the modulation of the miR-7a-5p/α-syn pathway in PD (Liu et al., 2022). Moreover, circ_0004381 functions as a sponge of miR-185-5p to affect RAC1 expression, contributing to MPP(+)-triggered neuron injury in a cellular model of PD in SK-N-SH cells (Zhang et al., 2022).

### Traumatic brain injury

Due to the higher rates of morbidity and mortality, TBI with unclear mechanisms is a serious problem for individuals and society. In the hippocampus of TBI rats, the expressions of circ_006508 and circ_010705 are upregulated, while the expressions of circ_001167 and circ_001168 are downregulated (Xie et al., 2018). In C57BL/6 mice subjected to TBI, increased circRNA chr8_87859283-87904548 blocks the restoration of neurological function after TBI by the chr8_87859283-87904548/mmu-let-7a-5p/CXCR2 axis (Chen et al., 2019). According to the profile of exosomes isolated from the cerebral extracellular space after TBI, 231 significantly dysregulated circRNAs (155 upregulated and 76 downregulated) were on presentation. Pathways of neuronal growth and repair, development, glutamatergic synapse signal transmission, and cyclic guanosine monophosphate–protein kinase G were predicted according to the Kyoto Encyclopedia of Genes and Genomes (KEGG) and Gene Ontology (GO) analyses (Zhao et al., 2018).

Circ_0020269 (circHtra1) was significantly upregulated in the brain with TBI. CircHtra1 serves as a miR-3960 sponge, upregulates the levels of GRB10, inhibits cell proliferation, and promotes neuronal apoptosis and NK cell infiltration after TBI (Zheng et al., 2022). After the validation of the polymerase chain reaction (PCR) of 10 circRNAs, circ_009194 was the most upregulated in the hippocampus after TBI. Mechanistically, circ_009194 functions as a sponge for miR-145-3p to regulate Sp1-mediated voltage-gated sodium channel (Nav1.3) in neurological outcomes in TBI (Huang X. J. et al., 2022). circ_010705 (circLrp1b) is significantly upregulated in the brain with TBI induced by controlled cortical impact (CCI). Upregulated circLrp1b as a sponge of miR-27a-3p increases the expression of Dram2 and promotes neurologic impairment, autophagy, and inflammation after TBI (Li H. et al., 2020).

### Stroke

Cerebrovascular disease with complicated mechanisms is one of the top three causes of disability-adjusted life-years (DALYs) globally (Collaborators GDH, 2017). The expression of circRNA circ_008018, circ_015350, and circ_016128 was upregulated, while the expression of circ_011137, circ_001729, and circ_006696 was downregulated. Each of these validated circRNAs may have more than 60 binding sites for miRNAs, which may be involved in the predicted pathways of MAPK, the cell cycle, and the regulation of the actin cytoskeleton (Mehta et al., 2017). In ischemic brain stroke, three (1,027 in total) circRNAs (circ_40001, circ_013120, and circ_40806) with the corresponding pathway of Rap1 signaling and Hippo signaling (according to the KEGG and GO analysis) regulate cell survival, death, and recovery through networks of circRNA–miRNA-target genes (Liu et al., 2017). However, the mechanism of circRNAs in the development of stroke remains largely unknown.

Expressions of circRNA Hectd1 (circHectd1) in ischemic stroke tissues from transient middle cerebral artery occlusion (tMCAO) mice and in plasma samples from patients with acute ischemic stroke (AIS) are significantly increased. In tMCAO mice, the knockdown of circHectd1 can significantly decrease cerebral infarct size, neuronal deficits, and astrocyte activation, though miR-142 targets TCDD inducible poly[ADP-ribose] polymerase (TIPARP) and inhibits the astrocyte activation *via* macroautophagy/autophagy (Han et al., 2018). The levels of circHectd1 are increased in patients with AIS, predicting a much higher risk of AIS recurrence with an area under the curve (AUC) of 0.694 (95% confidence interval (CI): 0.586–0.801) (Peng et al., 2019). The downregulation of circHectd1 induces neuroprotection against ischemic stroke through the miR-133b/TRAF3 pathway (Dai et al., 2021). CircHectd1 regulates ischemic stroke injury *via* mechanisms involving the regulation of the let-7c-5p/ROCK1 axis (Guo et al., 2022).

Injection of circDLGAP4 can significantly attenuate neurological deficits, infarct size, and blood–brain barrier damage *via* circDLGAP4/miR-143/*Hectd1* axes in tMCAO in a mouse stroke model (Bai et al., 2018). Bioinformatic analysis indicates that the upregulation of circ_015947 could enhance the expression of the predicted sponging miRNAs (miR-188-3p, miR-329-5p, miR-3057-3p, miR-5098, and miR-683), which are involved in apoptosis, metabolism, and the immune-related pathways in the pathogenesis of stroke (Lin et al., 2016). Circ_0025984 ameliorates ischemic stroke injury and protects astrocytes *via* the miR-143-3p/TET1/ORP150 pathway (Zhou et al., 2021). Overexpression of circ_0000831 is sufficient to inhibit neuroinflammation and vertigo in cerebral ischemia through a miR-16-5p-dependent mechanism (Huang et al., 2022). circSKA3, in an area under the ROC curve of 0.614 (95% CI: 0.546–0.680) in predicting clinical outcomes of patients with AIS, acts as a sponge of miR-6796-5p by regulating the expression of matrix metalloproteinase 9 (Xu T. et al., 2022). Hypoxic pretreated adipose-derived stem cell (ADSC) exosome improves cognitive function by decreasing neuronal damage and shifting microglia from an M1 to M2 phenotype in the hippocampus of AIS *via* circRps5/miR-124-3p/SIRT7 axes (Yang H. et al., 2022). CircUSP36 attenuates ischemic stroke injury through the miR-139-3p/SMAD3/Bcl2 signal axis (Yang J. et al., 2022). The ratio of serum circRNA-284 to miR-221 may serve as a diagnostic biomarker of carotid plaque rupture and stroke with an AUC of 0.98 (95% CI: 0.96–1.00) (Bazan et al., 2017). As mentioned above, several circRNAs function as both potential therapeutics and biomarkers for ischemic stroke.

### Postoperative neurocognitive disorder

During postoperative recovery, patients suffer from neurocognitive disorders. Preclinical neurocognitive disorders before anesthesia and surgery, the early onset of postoperative delirium, and long-lasting PONDs fall under the recommended terminology “perioperative neurocognitive disorders” (Evered et al., 2018). POND is defined by the presence of impaired memory, learning, and executive function after surgery (Migirov et al., 2021; Peden et al., 2021). POND without effective therapeutics and clear mechanisms is becoming a problem due to increased mortality, prolonged hospitalization, reliance on social transfer payments, and decreased quality of life (Eckenhoff et al., 2020; Peden et al., 2021).

The roles of ncRNAs in the development of POND are mentioned in our recent review elsewhere (He et al., 2021). Recent studies demonstrated that circ_089763 may be a crucial circRNA and function as a biomarker in the development of POND (Wang et al., 2019; Gao et al., 2020; Zhou et al., 2020). In serum samples from elderly patients with POND, the expression of circ_061570, circ_001145, and circ_101138 is increased (Gao et al., 2020). However, the mechanisms and effective therapeutics are unclear. ITSN1, a parent gene of circ_061570, plays an important role in early endocytic anomalies and the incidence of AD (Keating et al., 2006; Yu et al., 2008). Circ_001145 can sponge miR-1226-5p to the target gene Itsn1 in terms of circRNA–miRNA–mRNA axes. The network of circ_101138/miR-107/NEDD9 is predicted by Gao et al. (2020). Also, the upregulation of circ_009789 and circ_004229 may act as a sponge of miR-298-5p to upregulate the expression of Prkcb and Zbtb4 in the hippocampus of aging mice. Here, PKC signaling pathway, neural cell apoptosis, and glycolipid metabolism pathway are involved in the development of working memory dysfunction after surgery (Zhang M. X. et al., 2022).

Additionally, circ_101138 could regulate miR-376a/b-3p targeting HAS2 (Gao et al., 2020), which was associated with the pathogenesis of neuropathologic changes (Reed et al., 2019) and tau protein in AD-related neurocognitive dysfunction (Li et al., 2017). Additionally, circ_101138 could regulate miR-107, which was downregulated and a marker of the neurodegenerative process in AD concerning the Aβ metabolism and the inordinate cell cycle (Prendecki et al., 2019). However, the downregulation of miR-107 exerts the upregulation of SYK and worsens spatial memory in AD mice by activating the NF-κB signaling pathway (Hu et al., 2019). Here, circ_101138/miR-107/Syk might be another network of circRNA–miRNA–mRNA in the development of both POND and AD. A network of circ_101138/miR-107/Syk may explain the changes of POND in AD.

In another cohort study on geriatrics with no previous diagnosis of dementia, the level of Aβ1-42 in cerebral spinal fluid was the strongest independent predictor of postoperative delirium (included in PONDs) after elective arthroplasty in an aging population (Cunningham et al., 2019). Aβ is one of the key molecules involved in neurodegenerative diseases, and its degradation is partially regulated by the aforementioned networks of ciRS-7/miR-7a/Uchl1 and ciRS-7/miR-7a/Ubea2 (Zhao et al., 2016; Shi et al., 2017). As mentioned above, circHDAC9/miR-138/Sirt1 was involved in AD (Lu et al., 2019), while a decrease in Sirt1 was associated with improved postoperative cognition after cardiac surgery (Shi et al., 2020). In addition, circRNAs were engaged against POND by dexmedetomidine (Cao et al., 2020). Thus, the potential and protective roles of the circRNA–miRNA–mRNA axes would be studied in the future.

## Conclusions and perspectives

**Figure 2:**
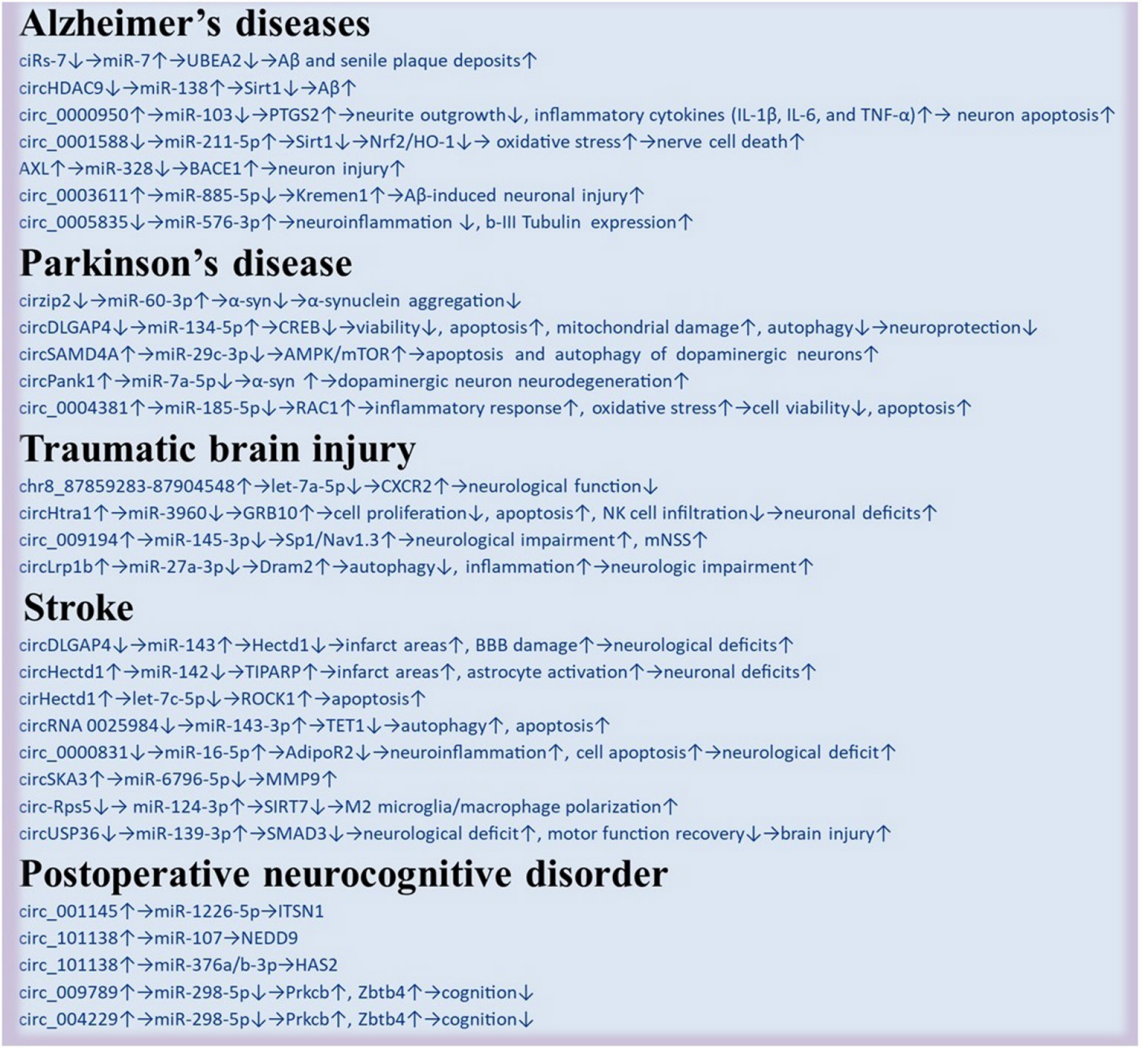
Regulatory networks of circRNA–miRNA–mRNA in various brain disorders. Changes in the regulatory networks of circRNA–miRNA–mRNA and potential signaling pathways are presented.

The crosstalk between circRNA, miRNA, and mRNA (or proteins) through one or more circRNA–miRNA–mRNA networks indicates the complicated and dynamic mechanisms of brain cognitive disorders including AD (Zhao et al., 2016; Lu et al., 2019; Yang et al., 2019; Zhu et al., 2020; Li et al., 2022; Pan et al., 2022; Xu X. et al., 2022), PD (Kumar et al., 2018; Feng et al., 2020; Wang et al., 2021; Liu et al., 2022; Zhang et al., 2022), TBI (Chen et al., 2019; Li H. et al., 2020; Huang X. J. et al., 2022; Zheng et al., 2022), stroke (Bai et al., 2018; Han et al., 2018; Zhou et al., 2021; Guo et al., 2022; Huang et al., 2022; Xu T. et al., 2022; Yang H. et al., 2022; Yang J. et al., 2022), and POND (Gao et al., 2020) (Table 1). In summary, circRNAs act as sponging-like roles in transcription and posttranscription in the novel regulation processes of gene expression. However, none of the aforementioned studies were able to clearly determine the exact role of circRNA–miRNA–mRNA axes in the pathogenesis and development of brain disorders with cognitive dysfunction or impairment (Figure 2).

**Table 1:** Circular RNAs (CircRNAs) in various brain cognitive disorders.

**Diseases/** **conditions**	**circRNAs**	**Sources (species, tissue, cells)**	**Trend**	**Roles**	**Functions**	**References**
AD	ciRs-7	Temporal lobe and hippocampal CA1 in 6 AD patients	Down	Promotor	ciRs-7↓ → miR-7↑ → UBEA2↓ → Aβ and senile plaque deposits↑	(Zhao et al., 2016)
AD	circHDAC9	Patients (5 males, 2 females); male APP/PS1 transgenic mice	Down	Suppressor	circHDAC9↓ → miR-138↑ → Sirt1↓ → Aβ↑	(Lu et al., 2019)
AD	circ_0000950	PC12 cells; rat cerebral cortex neurons	Down	Promotor	circ_0000950↑ → miR-103↓ → PTGS2↑ → neurite outgrowth↓, inflammatory cytokines (IL-1β, IL-6, and TNF-α)↑ → neuron apoptosis↑	(Yang et al., 2019)
AD	circ_0001588	Male Wistar rats; Neuro-2a cell line	Down	Suppressor	circ_0001588↓ → miR-211-5p↑ → Sirt1↓ → Nrf2/HO-1↓ → oxidative stress↑ → nerve cell death↑	(Zhu et al., 2020)
AD	AXL	SK-N-SH and SK-SY5Y cells	Up	Promotor	AXL↑ → miR-328↓ → BACE1↑ → neuron injury↑	(Li et al., 2022)
AD	circ_0003611	SK-N-SH cells	Up	Promotor	circ_0003611↑ → miR-885-5p↓ → Kremen1↑ → Aβ-induced neuronal injury↑	(Pan et al., 2022)
AD	circ_0005835	Thirty AD patients; SH-SY5Y and BV2 cells	Up	Promotor	circ_0005835↓ → miR-576-3p↑ → neuroinflammation ↓, b-III Tubulin expression↑	(Xu X. et al., 2022)
**PD**	cirzip2	*C*. *elegans* Bristol strain N2 and NL5901 strain	Down	Promotor	cirzip2↓ → miR-60-3p↑ → α-syn↓ → α-synuclein aggregation↓	(Kumar et al., 2018)
**PD**	circDLGAP4	MPTP-induced mouse; MPP-induced SH-SY5Y and MN9D cells	Down	Suppressor	circDLGAP4↓ → miR-134-5p↑ → CREB↓ → viability↓, apoptosis↑, mitochondrial damage↑, autophagy↓ → neuroprotection↓	(Feng et al., 2020)
**PD**	circSAMD4A	MPTP-induced mouse; MPP-induced SH-SY5Y cells	Up	Promotor	circSAMD4A↑ → miR-29c-3p↓ → AMPK/mTOR↑ → apoptosis and autophagy of dopaminergic neurons↑	(Wang et al., 2021)
**PD**	circPank1	Mice; MN9D cells	Up	Promotor	circPank1↑ → miR-7a-5p↓ → α-syn ↑ → dopaminergic neuron neurodegeneration↑	(Liu et al., 2022)
**PD**	circ_0004381	MPP-induced SK-N-SH cells	Up	Promotor	circ_0004381↑ → miR-185-5p↓ → RAC1↑ → inflammatory response↑, oxidative stress↑ → cell viability↓, apoptosis↑	(Zhang et al., 2022)
TBI	chr8_87859283-87904548	C57BL/6 mice	Up	Promotor	chr8_87859283-87904548↑ → let-7a-5p↓ → CXCR2↑ → neurological function↓	(Chen et al., 2019)
TBI	circHtra1	Hippocampus of rat	Up	Promotor	circHtra1↑ → miR-3960↓ → GRB10↑ → cell proliferation↓,apoptosis↑, NK cell infiltration↓ → neuronal deficits↑	(Zheng et al., 2022)
TBI	circ_009194	Hippocampus of rat	Up	Promotor	circ_009194↑ → miR-145-3p↓ → Sp1/Nav1.3↑ → neurological impairment↑, mNSS↑	(Huang X. J. et al., 2022)
TBI	circLrp1b	Hippocampus of rat	Up	Promotor	circLrp1b↑ → miR-27a-3p↓ → Dram2↑ → autophagy↓, inflammation↑ → neurologic impairment↑	(Li H. et al., 2020)
Stroke	circDLGAP4	Patients (13 females, 13 males); mice	Down	Suppressor	circDLGAP4↓ → miR-143↑ → Hectd1↓ → infarct areas↑, BBB damage↑ → neurological deficits↑	(Bai et al., 2018)
Stroke	Hectd1	Plasma from AIS patients; tMCAO mice	Up	Promotor	circHectd1↑ → miR-142↓ → TIPARP↑ → infarct areas↑, astrocyte activation↑ → neuronal deficits↑	(Han et al., 2018)
Stroke	Hectd1	PC12 cells with OGD/R	Up	Promotor	cirHectd1↑ → let-7c-5p↓ → ROCK1↑ → apoptosis↑	(Guo et al., 2022)
Stroke	circ_0025984	Male SD rats with MCAO	Down	Suppressor	circRNA_0025984↓ → miR-143-3p↑ → TET1↓ → autophagy↑, apoptosis↑	(Zhou et al., 2021)
Stroke	circ_0000831	MCAO in mice	Down	Suppressor	circ_0000831↓ → miR-16-5p↑ → AdipoR2↓ → neuroinflammation↑, cell apoptosis↑ → neurological deficit↑	(Huang et al., 2022)
Stroke	circSKA3	Ischemic stroke patients (*n* = 220)	Up	Promotor	circSKA3↑ → miR-6796-5p↓ → MMP9↑	(Xu T. et al., 2022)
Stroke	circRps5	MCAO in mice	Down	Suppressor	circRps5↓ → miR-124-3p↑ → SIRT7↓ → M2 microglia/macrophage polarization↑	(Yang H. et al., 2022)
Stroke	circUSP36	Ischemic stroke patients; tMCAO mice	Down	Suppressor	circUSP36↓ → miR-139-3p↑ → SMAD3↓ → neurological deficit↑, motor function recovery↓ → brain injury↑	(Yang J. et al., 2022)
POND	circ_001145	Patients (*n* = 3)	Up	unknown	circ_001145↑ → miR-1226-5p → ITSN1*	(Gao et al., 2020)
POND	circ_101138	Patients (*n* = 3)	Up	unknown	circ_101138↑ → miR-107 → NEDD9*	(Gao et al., 2020)
POND	circ_101138	Patients (*n* = 3)	Up	unknown	circ_101138↑ → miR-376a/b-3p → HAS2*	(Gao et al., 2020)
POND	circ_009789	Aging mice brain; Neuro-2a cells	Up	Promotor	circ_009789↑ → miR-298-5p↓ → Prkcb↑, Zbtb4↑ → cognition↓	(Zhang M. X. et al., 2022)
POND	circ_004229	Aging mice brain; Neuro-2a cells	Up	Promotor	circ_004229↑ → miR-298-5p↓ → Prkcb↑, Zbtb4↑ → cognition↓	(Zhang M. X. et al., 2022)

Expressions of circRNAs mentioned above were determined by real-time quantitative polymerase chain reaction (RT-PCR). ^*^Predicted networks only.

AD, Alzheimer's disease; Aβ, β-amyloid; APP, β-amyloid precursor protein; BACE1, β-site APP-cleaving enzyme 1; NF-κB, nuclear factor kappa B; UBE2A, ubiquitin conjugating enzyme E2A; UCHL1, ubiquitin carboxyl-terminal hydrolase L1; Sirt1, silent information regulator 1; Nfr2, nuclear factor erythroid 2-related factor 2; HO-1, heme oxygenase-1; PTGS2, prostaglandin-endoperoxide synthase 2; PD, Parkinson's disease; TIPARP, TCDD inducible poly(ADP-ribose) polymerase; BBB, blood–brain barrier; tMCAO, transient middle cerebral artery occlusion; AIS, acute ischemic stroke; OGD/R, oxygen-glucose deprivation/reoxygenation; ROCK1, Rho-associated coiled-coil-containing protein kinase 1; MMP9, matrix metalloproteinase 9; TBI, traumatic brain injury; mNSS, modified Neurological Severity Score; ADSCs, adipose-derived stem cells; POND, postoperative neurocognitive disorder.

Different circRNAs sponge different miRNAs under various brain disorders (Figure 2). Meanwhile, the same circDLGAP4 can be involved in sponging different miRNAs, such as miR-143 in stroke (Bai et al., 2018) and miR-134-5p in PD (Feng et al., 2020). As it indicates that similar circRNA sponging different miRNAs may occur in different diseases. However, in similar brain disorders or conditions, circHectd1 can sponge different miRNAs, including miR-133b, let-7c-5p, and miR-142 (Han et al., 2018; Dai et al., 2021; Guo et al., 2022), indicating that one disease may embrace different pathways under different conditions. However, how should we check and determine which is the best pathway (Gao et al., 2020)?

The aforementioned studies on the circRNA–miRNA–mRNA network suggest that circRNAs as endogenous RNAs (ceRNAs) play important roles in discovering the pathogenesis and development of brain disorders in the future. Recently, some studies indicate that a subset of circRNAs has been considered as codes for proteins (Ma et al., 2020; Sinha et al., 2021). However, the available evidence shows that circRNA translation is not an efficient event. Herein, many more studies on circRNA–miRNA–mRNA networks are needed to update and expand our knowledge about increasing effective strategies to protect individuals from these brain disorders.

## Author contributions

Conceptualization, writing—original draft, and funding acquisition: LH. Formal analysis: LH, FZ, and YZ. Writing—review and editing: LH and ML. All authors had full access to all the data in the study and take responsibility for the integrity of the data and the accuracy of the data analysis, contributed to the article, and approved the submitted version.

## Funding

This work was supported by the Chuncheng Youth Top-notch Talent Support Program (2020), the Health and Family Planning Commission of Yunnan province (H-2017047), and partially by the National Natural Science Foundation of China (81860208).

## Conflict of interest

The authors declare that the research was conducted in the absence of any commercial or financial relationships that could be construed as a potential conflict of interest.

## Publisher's note

All claims expressed in this article are solely those of the authors and do not necessarily represent those of their affiliated organizations, or those of the publisher, the editors and the reviewers. Any product that may be evaluated in this article, or claim that may be made by its manufacturer, is not guaranteed or endorsed by the publisher.
